# Prebiotic and Synbiotic Modifications of Beta Oxidation and Lipogenic Gene Expression after Experimental Hypercholesterolemia in Rat Liver

**DOI:** 10.3389/fmicb.2017.02010

**Published:** 2017-10-17

**Authors:** Claudia C. Alves, Dan L. Waitzberg, Laila S. de Andrade, Laís dos Santos Aguiar, Milene B. Reis, Camila C. Guanabara, Odair A. Júnior, Daniel A. Ribeiro, Priscila Sala

**Affiliations:** ^1^Department of Biosciences, Federal University of São Paulo, Santos, Brazil; ^2^Department of Gastroenterology, School of Medicine, University of São Paulo, São Paulo, Brazil; ^3^Nutrition Graduate, Federal University of São Paulo, Santos, Brazil

**Keywords:** cholesterol diet, steatosis, rats, prebiotic, probiotic, synbiotic, gene expression

## Abstract

**Background and aims:** Non-alcoholic fatty liver disease (NAFLD) is characterized by the presence of fat in hepatocytes because of decreased β-oxidation and increased lipogenesis. Prebiotics, probiotics, and synbiotic have modulatory effects on intestinal microbiota and may influence the gut-liver axis. Our aim was to evaluate the effects of prebiotic, probiotics, and synbiotic on liver histopathology and gene expression related to β-oxidation and lipogenesis after hypercholesterolemia.

**Methods:** Wistar male adult rats (*n* = 40) were submitted to hypercholesterolemic conditions (HPC) (60 days). On Day 30 of HPC, rats were subdivided in 5 groups: negative control (NC): without HPC + Gv (distilled water); positive control (PC): with HPC + Gv (distilled water); prebiotic (PRE): HPC + Gv with prebiotic (Fiber FOS^®^); probiotic (PRO): HPC + Gv with probiotic strains Gv (Probiatop^®^); and synbiotic (SYN): HPC + Gv with synbiotic (Simbioflora^®^). All rats were sacrificed on Day 30 post-treatment. Blood was collected to verify total serum cholesterol, and liver tissue was sampled to verify histopathological changes and gene expression. Gene expression related to ß-oxidation (PPAR-α and CPT-1) and lipogenesis (SREBP-1c, FAS and ME) was evaluated in liver tissue using RT-qPCR.

**Results:** PC had higher cholesterol levels when compared to NC. PRE and SYN rats had lower cholesterol levels than PC. PC rats showed more histopathological changes than NC rats; PRE and SYN rats showed fewer alterations than PC rats. *PPAR-α* was expressed at higher levels in SYN and PC rats compared with PRE and PRO rats. *CPT-1* expression was similar in all groups. *SREBP-1c* was expressed at higher levels in PC rats compared with NC rats; levels were lower in SYN rats compared with PRO rats; levels were lower in PRE rats compared with PC and PRO rats. *FAS* was expressed at lower levels in PRE rats compared with SYN rats. *ME* expression was lower in PC rats compared with NC rats.

**Conclusion:** Prebiotic and synbiotic supplementation improve hepatic alterations related to hypercholesterolemia. These changes appear to be mediated by altered expression of genes related to β-oxidation and lipogenesis.

## Introduction

Non-alcoholic fatty liver disease (NAFLD) can be understood as the excessive accumulation of fat in hepatocytes, without alcohol abuse (Buzzetti et al., 2016). Currently, NAFLD is a major cause of chronic liver disease in Western countries, and its prevalence has increased in other countries because of globalization, which increases the consumption of highly processed and refined foods (Younossi et al., 2016). NAFLD is associated with metabolic disorders such as obesity, type 2 diabetes mellitus, and dyslipidemia (Lonardo et al., 2015).

Clinically, the treatment of NAFLD is very difficult to achieve for most patients. In this regard, to search genetic biomarkers closely involved to NAFLD etiopathogenesis is important for better understanding the disease (Reddy, 2001; Ahmed and Byrne, 2007). Hepatic steatosis in NAFLD results from hepatic lipid accumulation due to decreased β-oxidation of fatty acids and increased free fatty acids in the liver, which contribute to hepatic lipogenesis (Donnelly et al., 2005). The triggering of β-oxidation involves coordinated gene expression regulated by nuclear receptors, such as peroxisome-proliferator activated receptors (PPARs). In particular, PPAR-α is expressed primarily in tissues rich in mitochondria, such as liver. It is considered an important factor regulating the β-oxidation of fatty acids (Reddy, 2001) that prevents the excessive storage of liver fat (Stienstra et al., 2007). One of the limiting factors in β-oxidation is the presence of carnitine palmitoyl transferase enzyme 1 (CPT-1), which determines the intensity of hepatic mitochondrial β-oxidation (Donnelly et al., 2005).

Cholesterol and fatty acids levels are regulated through distinct pathways, but these different patterns of regulation are controlled by a common family of transcription factors denominated sterol regulatory element-binding proteins (SREBPs). They regulate hepatic cholesterol homeostasis. SREBP-1c is the main modulator of hepatic metabolism of glucose and triglycerides; this activity may be involved in lipid accumulation in NAFLD. It operates in the activation of lipogenic genes such as fatty acid synthase (FAS) and malic enzyme (ME) (Ahmed and Byrne, 2007).

There is a functional link between the gut and liver called gut-liver axis. Factors as intestinal microbiota, barrier function and immune responses play an important role in gut-liver axis (Minemura and Shimizu, 2015). Altered gut microbiota (dysbiosis) may contribute to many diseases in local and remote organ systems. Intestinal and extra-intestinal diseases, including liver disease, impacts the normal function of microbiota enhancing the permeability and endotoxin translocation (Fukui, 2015; Minemura and Shimizu, 2015; Seki and Schwabe, 2017). The intestinal microbiota has a symbiotic interaction with all human body functions (Minemura and Shimizu, 2015). Thus, nutritional interventions may be useful to corroborate with the treatment of liver disease by modulating gut microbiota. Prebiotics are non-digestible carbohydrates such as fructooligosaccharides (Leung et al., 2016). When fermented by colonic bacteria, prebiotics stimulate the proliferation or activity of probiotic bacteria that support the function of the host intestine and prevent the proliferation of pathogenic bacteria (Guarner et al., 2012). Probiotics are live microorganisms such as lactobacilli and bifidobacteria; when consumed by humans in sufficient amounts, these microorganisms confer a health benefit on the host (FAO and WHO, 2017). The combination of prebiotic fiber with probiotic bacteria results in a synergistic compound known as “synbiotic” (Guarner et al., 2012).

Accumulating evidence suggests that probiotics, prebiotics, and synbiotic may support intestinal microbiota, improve lipid metabolism, and contribute to the treatment of liver disease by influencing the intestine-liver axis (Shibolet et al., 2002; Esposito et al., 2009; Kok et al., 2017). It is important to know, in NAFLD, which histological effects and molecular mechanisms may be altered by the use of biotics. The aim of our experimental study was to evaluate, in hypercholesterolemic rats, the effects of prebiotic, probiotic, and synbiotic supplementation on histopathology and gene expression related to β-oxidation and lipogenesis in liver tissue.

## Materials and Methods

All experiments protocols involving animals conformed to the procedures described in the Guiding Principles for the Use of Laboratory Animals. The study protocol was approved by the Animal Committee of Federal University of São Paulo, UNIFESP, SP, Brazil (Protocol number 1265/10).

### Experimental Design

A total of 40 male Wistar rats, weighing approximately 350 g, were obtained from the Centro de Desenvolvimento de Modelos Experimentais (CEDEME), Federal University of São Paulo, SP, Brazil. They were maintained under controlled conditions of temperature (24 ± 2°C) under 12 h light-12 h dark cycles, with free access to water and commercial diet (Nuvital, Paraná, Brazil). All animals were acclimatized for 10 days before the experiment and provided with *ad libitum* access to standard pellet chow and fresh water.

#### Hypercholesterolemic Condition

After acclimatization, all rats (except for the negative control group [NC]) were fed a cholesterol-enriched diet for 60 days to induce hypercholesterolemia. The NC group received standard oral rat chow (23% protein, 11% lipid, 66% carbohydrate) (Nuvilab^®^ Nuvital Ltda, Paraná, Brazil). To induce hypercholesterolemia, animals received the same standard chow added 1% (w/w) cholesterol (C8503; Sigma Chemical Company, St Louis, MO, United States) and 0.35% (w/w) cholic acid (C1254; Sigma Chemical Company) (Manzoni et al., 2005).

#### Nutritional Supplementation

On Day 30 of the hypercholesterolemic condition (HPC), rats (*N* = 40) were subdivided in 5 groups. Each group received a special diet by gavage (Gv). NC rats (*n* = 5) received only distilled water. Positive control (PC) (*n* = 5) received HPC and distilled water by Gv. Prebiotic (PRE) (*n* = 10) rats received 3 g/day of fructooligosaccharide (Fiber Fos^®^ Invictus Farmanutrição, FQM, São Paulo/Brazil), diluted in distilled water, by Gv. Probiotic (PRO) (*n* = 10) rats received 10^9^ CFU of each probiotic strain (*Lactobacillus paracasei* Lpc-37^®^ SD 5275^®^, *Lactobacillus rhamnosus* HN001^®^ SD 5675^®^, *Lactobacillus acidophilus* NCFM^®^ SD 5221^®^, *Bifidobacterium lactis* HN019^®^ SD 5674^®^; Probiatop^®^ Invictus Farmanutrição, FQM, São Paulo/Brazil) diluted in distilled water and administered by Gv. Synbiotic (SYN) (*n* = 10) rats received 3 g/day of fructooligosaccharide + 10^9^ CFU of each probiotic strain (*Lactobacillus paracasei* Lpc-37^®^ SD 5275^®^, *Lactobacillus rhamnosus* HN001^®^ SD 5675^®^, *Lactobacillus acidophilus* NCFM^®^ SD 5221^®^, *Bifidobacterium lactis* HN019^®^ SD 5674^®^; Simbioflora^®^ Invictus Farmanutrição, FQM, São Paulo, Brazil), diluted in distilled water and administered by Gv. All supplements were diluted in the same amount of distilled water (1.5 mL) and prepared fresh twice a day. All were administered twice a day by gavage. Food and water intake were verified three times a week throughout the experimental period.

#### Oral Gavage Technique

The oral gavage procedure was performed twice a day. A feeding needle (3-mm diameter) was attached to the syringe (5 mL) and placed into the right lateral side of the oral cavity. The needle was gently inserted into the back of the oral cavity and slipped down into the esophagus. The solution containing the different products (prebiotic, probiotic, or synbiotic solution or distilled water) was injected slowly, to prevent esophageal reflux into the oral cavity or rupture of the esophagus. The needle was then gently removed, and the animal was returned to its cage.

#### Body Weight during Nutritional Supplementation

Body weight was verified three times a week during the period of nutritional supplementation (30 days). Body weight was verified and recorded based on the initial (first day of supplementation period) and final (day of sacrifice) for each experimental group.

#### Liver Sampling

At the end of the supplementation period, rats were sacrificed with 0.4% sodium pentobarbital (1 ml/kg, intraperitoneal). Liver samples were longitudinally bisected for morphological examination, promptly identified, quickly fixed in 10% buffered formalin (Merck, Darmstadt, Germany), and embedded in paraffin blocks.

#### Measurement of Cholesterol Levels

At sacrifice, blood was collected by heart puncture for measurement of total plasma cholesterol. Measurements were obtained with the fast-color method using reagents from the Sera Pak-Ames-Analyzer (RZXT-Technicon, Ames, IA, United States).

#### Tissue Processing

After euthanasia, liver was removed from all animals. The tissues were fixed in 10% buffered formalin (Merck^TM^, Darmstadt, Germany), embedded in paraffin blocks and stained with hematoxylin and eosin (H&E., Merck^TM^, Darmstadt, Germany) for evaluating histopathological analysis.

#### Histopathological Analysis

Histopathological analysis was evaluated by light microscopy in a blind manner for two experienced observers (OA and DAR). For this purpose, it was observed the presence or absence of inflammatory cells, tissue degeneration, and necrosis, per animal. Digital images were taken from three to four H&E-stained sections derived from five fields per animal using An Olympus BX50 bright field microscope and a DP71 camera (Melville, NY, United States) with a 40x objective. A semi-quantitative method was used for evaluating the histopathological alterations as described in **Table 1** according to Zhang et al. (2006).

**Table 1:** Scoring system for histopathological alterations (Zhang et al., 2006).

Score	Steatosis area (%)	Inflammation
0	0	0
1	<30	Watering degeneration and some small necrosis
2	30–50	Ballooning degeneration, more small necrosis, Mallory body and local PMN infiltration
3	>50	Severe degeneration, necrosis, and bridging necrosis

*PMN, polymorphonuclear leukocyte.*

#### Total RNA Isolation and cDNA Synthesis

Total RNA was obtained from liver tissues using TRIzol^®^ Reagent (Life Technologies^®^, Carlsbad, CA, United States), according to the manufactures instructions. Samples were treated with DNA se (Life Technologies^®^, Carlsbad, CA, United States) to avoid contamination with genomic DNA. RNA concentrations were measured with a NanoDrop ND-1000 spectrophotometer (NanoDrop Technologies, Wilmington, DE, United States).

Reverse transcription of 1 μg total RNA to cDNA was performed in a Veriti^TM^ PCR Thermal Cycler (Applied Biosystems, Foster City, CA, United States) using a High-Capacity cDNA Reverse Transcription Kit (Applied Biosystems, Foster City, CA, United States).

#### Real-Time Quantitative Polymerase Chain Reaction (RT-qPCR)

RT-qPCR amplification was performed in an Applied Biosystems 7500 FAST Real Time PCR system (Applied Biosystems, Foster City, CA, United States) thermal cycler using TaqMan^TM^Gene Expression Master Mix and Assays (Applied Biosystems, Foster City, CA, United States). The following TaqMan^TM^ assays were purchased and used: PPAR-α (Rn 00566193_m1), CPT-1 (Rn 00580702_m1), SREBP-1 (Rn 01495769_m1), FAS (Rn 01463550_m1), ME (Rn 00667869_m1), and GAPDH (Rn 01775763_g1; endogenous control). The following thermal cycling conditions were used: 50°C for 2 min (activation step), 95°C for 10 min (polymerase activation step), followed by forty cycles of 95°C for 15 s (denaturation) and 60°C for 1 min (annealing/extension). Relative amounts of target mRNA were calculated using the comparative cycle threshold (Ct) method described by Pfaffl (2001).

### Statistical Methods

Statistical analysis for body weight, total cholesterol and RT-qPCR values was performed with one-way ANOVA followed by Tukey’s test. Histopathological scores were assessed with the Fisher test, using the SPSS software package (version 1.0; SPSS Institute, Chicago, IL, United States). *P* < 0.05 was considered statistically significant.

## Results

### Clinical Findings

Use of the oral gavage technique resulted in only one death (SYN group), on Day 10 of the supplementation period. For all experimental groups evaluated, there were no differences between initial and final body weight during the nutritional supplementation period (*P* > 0.05; **Table 2**).

**Table 2:** Body weight and total plasma cholesterol levels.

Group	Initial body	Final body	Plasma total
(N)	weight (g)	weight (g)	cholesterol (ng/L)
NC (5)	356.60 ± 41.73	419.60 ± 37.29	90.80 ± 12.47
PC (5)	350.20 ± 39.66	400.00 ± 38.88	132.85 ± 29.29^∗^
PRE (10)	352.10 ± 29.14	395.80 ± 40.37	88.11 ± 8.39^#^
PRO (10)	348.67 ± 44.31	396.89 ± 35.65	119.55 ± 24.45
SYN (9)	344.22 ± 47.93	394.33 ± 39.17	99.05 ± 13.71^#^

*NC, negative control; PC, positive control; PRE, prebiotic group; PRO, probiotic group; SYN, synbiotic group. *Initial body weight - first day of supplementation period. Final body weight - day of sacrifice.* Plasma Total cholesterol: ^∗^PC > NC (*P* = 0.009); ^#^PRE and SYN < PC (P < 0.001).*

Rats in the PC group fed a cholesterol-enriched diet had elevated serum cholesterol compared with NC rats. PRE and SYN rats had lower cholesterol levels than PC rats (*P* < 0.05; **Table 2**).

**FIGURE 1:**
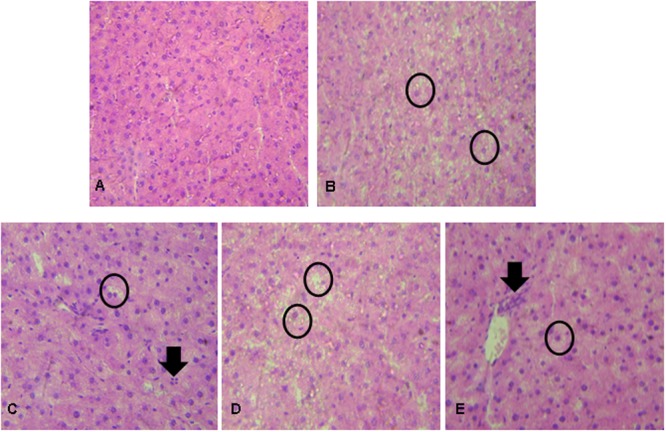
Liver histopathological analysis in rats after exposure to hypercholesterolemic conditions and prebiotic, probiotic, and/or synbiotic supplementation. **(A)** NC showing ordinary appearance; **(B)** PC (hypercholesterolemic diet) showing severe stetatosis in rat hepatocytes (Circle); **(C)** Prebiotic group showing the presence of mild steatosis in some rat hepatocytes (circle) and the presence of inflammatory cells (arrow); **(D)** Probiotic group showing severe steatosis in rat hepatocytes (circle); **(E)** Synbiotic group showing mild stetatosis in some rat hepatocytes (Circle) and the presence of inflammatory cells (arrow). Hematoxylin and eosin stain, 40×.

Histopathological changes in liver tissues varied among experimental groups. None of these changes resulted in a severe score following microscopic evaluation. Liver tissue from the PC group presented more hepatic degeneration and increased steatosis (**Figure 1B**) than observed in samples from the NC group (**Figure 1A**). Rats that received prebiotic and synbiotic supplementation had decreased steatosis (**Figures 1C,E**) compared with PC animals. No statistical difference in liver steatosis was observed when the PRO group (**Figure 1D**) was compared with the PC group (**Table 3**).

**Table 3:** Number of animals according to the degree of histopathological change in liver tissue, according Zhang et al. (2006).

Groups	Scores			
	0	1	2	3
NC	5	0	0	0
PC	0	3	2	0
PRE	6	2	0	0
PRO	5	2	1	0
SYN	5	3	0	0

*NC, negative control; PC, positive control; PRE, prebiotic group; PRO, probiotic group; SYN, synbiotic group.*

### Gene Expression Analysis

**FIGURE 2:**
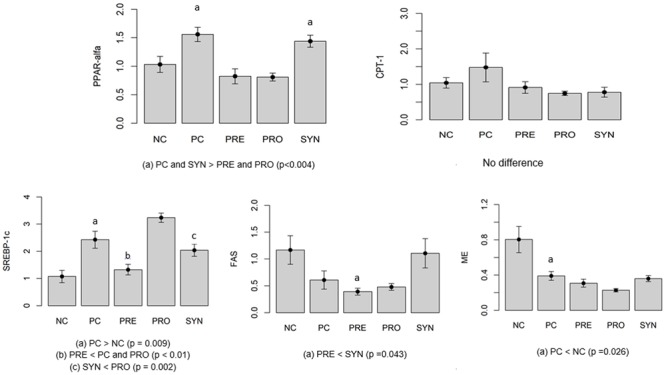
Gene expression indicating levels of ß-oxidation (*PPAR-α, CPT-1*) and lipogenesis (*SREBP-1c, FAS, ME*). Values expressed as arbitrary units (AU). All data expressed as means ± SED.

To investigate the β-oxidation process, we measured PPAR-α and CPT-1 gene expression (**Figure 2**). PPAR-α gene expression was higher in PC and SYN groups than in PRE and PRO groups (*P* < 0.004). CPT-1 levels in liver were similar in all experimental groups (*P* > 0.05).

Lipogenesis was evaluated by measuring gene expression levels of *SREBP-1c, FAS* and *ME* (**Figure 2**). *SREBP-1c* expression was higher in the PC group than in the NC group (*P* = 0.009). Levels were significantly lower in the SYN group compared with the PRO group (*P* = 0.002). *SREBP-1c* expression was lower in the PRE group compared with the PC and PRO groups (*P* < 0.01). To investigate lipogenesis, we measured FAS mRNA gene expression; levels were lower in the PRE group compared with the SYN group (*P* = 0.043). *ME* gene expression was lower in the PC group than in the NC group (*P* = 0.026).

## Discussion

Non-alcoholic fatty liver disease is increasingly prevalent and represents a challenge for prevention and treatment; the condition’s pathogenesis remains poorly understood. The goal of the present study was to evaluate the effects of prebiotic, probiotic and synbiotic treatment in rats submitted to HPCs. We evaluated the effects of treatment on serum cholesterol, histopathological changes in the liver, and gene expression related to ß-oxidation (PPAR-α, CPT-1) and lipogenesis (SREBP-1c, FAS, ME). To the best of our knowledge, the approach has not been addressed so far.

Previous experimental studies induced steatosis by increasing the level of saturated lipid in the diet (de Wit et al., 2012) or by altering the composition of dietary fat (i.e., providing high levels of unsaturated fat, lard or corn oil) (Nanji, 2004; Lieber et al., 2004; Esposito et al., 2009; Tipoe et al., 2009; Kok et al., 2017). In our experimental model, steatosis was obtained by providing a chow diet containing 1% cholesterol with 0.25% cholic acid supplementation, for 60 days (Rossi et al., 2000).

In rats, dyslipidemia may be prevented by mechanisms that reduce the synthesis of cholesterol and increase bile acid excretion, reducing the increase in serum cholesterol levels after ingestion of a diet rich in cholesterol (Lin and Connor, 1980). However, the addition of cholic acid to the diet favors the intestinal absorption of cholesterol and increases serum cholesterol (Guerra et al., 2007). In our experiment, PC animals fed a cholesterol-enriched diet showed a significant increase in serum cholesterol (46%) when compared to NC rats fed a standard oral chow diet. Under HPCs, rats developed characteristics of NAFLD, including increased serum cholesterol and histopathological changes in the liver.

Animal and human studies have confirmed a relationship between the gut microbiota and the pathogenesis of NAFLD (Tremaroli and Bäckhed, 2012; Moschen et al., 2013). The consumption of obesogenic foods such as fructose and lipid leads to changes in the gut microbiota and intestinal barrier, contributing to metabolic endotoxemia and inflammation (Cani et al., 2007). Germ-free mice are resistant to the development of obesity, steatosis, and insulin resistance (Bäckhed et al., 2007; Rabot et al., 2010).

The microbiota is essential for the better gut barrier function that controls the access of their products to the portal circulation and the liver (Minemura and Shimizu, 2015). Understanding the gut-liver axis it can lead to attempts to manipulate the microbiota. Probiotics, prebiotics and synbiotic may exert a role in the treatment and prevention on liver disease by reverting dysbiosis (Leung et al., 2016).

Although few studies have investigated the effects of gut microbiota on NAFLD, the data published to date suggest that pre-, pro- and synbiotic treatments are effective in modifying the intestinal microbiota and may be used as a therapeutic approach in NAFLD (Kirpich et al., 2015).

Our results showed that supplementation with prebiotics or synbiotics reduced serum cholesterol levels and histopathological changes when compared to control treatment. Arjmandi et al. (1992) previously found that rats fed fiber pectin (prebiotic fiber) had decreased levels of plasma cholesterol compared with the PC group. These results indicate a potential beneficial effect of fiber prebiotic intake on the regulation of blood cholesterol levels (Arjmandi et al., 1992).

Eslamparast et al. (2014) evaluated the effects of synbiotic supplementation on lifestyle in NAFLD patients. The synbiotic used contained 200 million CFU of 7 bacterial strains *(Lactobacillus casei, Lactobacillus rhamnosus, Streptococcus thermophilus, Bifidobacterium breve, Lactobacillus acidophilus, Bifidobacterium longum, Lactobacillus bulgaricus)* in combination with prebiotic fructooligosaccharide (20–30 g/day). The endpoint was improvement of hepatic inflammation and other liver functions. The results obtained after 28 days of synbiotic supplementation with lifestyle modification were superior to those obtained with lifestyle modification alone. This trend indicates the potential utilitiy of synbiotics in NAFLD treatment (Eslamparast et al., 2014).

In another experimental study, a specific multistrain cocktail (VSL#3) composed of *Streptococcus thermophilus* and several species of *Lactobacillus* and *Bifidobacteria* was administered to rats fed a high-fat diet (HFD). Serum cholesterol and triglyceride concentrations were higher in the rats fed a HFD as compared to those fed a standard diet. When VSL#3 was administered, cholesterol and triglyceride concentrations were lower in rats fed the HFD than in counterparts fed a standard diet (Esposito et al., 2009).

In our study, histopathological examination of the liver revealed that cholesterol-treated rats (PC) displayed extensive steatosis, liver degeneration with inflammatory cells, and necrosis, when compared to NC animals. Concomitant supplementation with a prebiotic and synbiotic was able to decrease the steatosis features induced by cholesterol, indicating a protective effect on the liver. We speculate that this effect occurred in PRE and SYN groups by mechanisms that connect gut microbiota and NAFLD such as: 1- fermentation of polysaccharides by microbiota into monosaccharides and short chain fatty acids (SCFAs), such as acetate, propionate, butyrate and ethanol SCFA serve as an energy source to host intestinal epithelium. Butyrate is the preferred energy substrate for colonocytes and its metabolism provides key substrates in cell metabolism. (Polyzos et al., 2009); 2- SCFA has been shown to reduce the permeability of tight junctions and/or the increased intestinal permeability may also direct SCFAs to access and modulate liver functions (Polyzos et al., 2017); 3- Decreased choline metabolism leading to decreased VLDL export from the liver (Polyzos et al., 2017); 4- Modulation of bile acid synthesis, which are crucial for fat absorption, but also affect metabolism of glucose and lipoproteins by linking farsenoid X receptor (FXR) (Verdelho Machado and Cortez-Pinto, 2017). Also, we emphasize that the better results in SYN group may be directed to prebiotic fiber (fructooligosaccharide) and not to probiotic strains containing in synbiotic product. Although Esposito et al. (2009) have documented positive effects for probiotic strains, our study failed to demonstrate this finding. Probably, these discrepancies could be explained by differing in experimental design. Further studies are welcomed to elucidate the issue, specially to clarify the role of symbioses on the activity of probiotic strains in living organisms.

Several factors may be involved in the pathogenesis of NAFLD genesis; the nutritional component is the main environmental factor linked with intestinal microbiota (Buzzetti et al., 2016). Nonetheless, few studies have been conducted to explain the interaction of NAFLD with intestinal microbiota and the intestine-liver axis being very difficult to establish deeper discussion on this matter (Kirpich et al., 2015).

Prebiotics and probiotics have numerous beneficial effects on body tissues and systems (Ulisse et al., 2001; Mach, 2006). Such effects may be mediated by mechanisms such as the modulation of local microbiota, epithelial barrier function, and the immune system (O’Hara and Shanahan, 2007). Because the modulatory effects on gut microbiota may influence the intestine-liver axis, the proliferation of non-pathogenic microorganisms could be used as adjunctive therapy in some cases of liver disease (Lirussi et al., 2007).

Lipid metabolism is a complex process that involves the coordinated expression of numerous genes. PPARs play a central role in the modulation of fatty acid oxidation and inflammation. PPAR-α is mainly expressed in tissues rich in mitochondria (e.g., liver) and is considered the primary factor regulating the β-oxidation of fatty acids (Reddy and Hashimoto, 2001), which limits the storage of liver fat (Stienstra et al., 2007). In our study, we found that PPAR-α expression was higher in the PC and synbiotic groups compared to rats that received supplementation with prebiotics and probiotics. The elevated levels of PPAR-α observed in the synbiotic group compared to the prebiotic and probiotic groups suggests that the simultaneous administration of probiotic and prebiotic (synbiotic) treatment may be more effective in increasing hepatic lipid oxidation. One reason for the lower levels of PPAR-α observed in the PC group may be that these animals were trying to metabolize the excessive amount of dietary fat, which required greater activation of this oxidation process. Esposito et al. (2009) found lower PPAR-α expression in rats fed a HFD compared with rats fed standard chow. The authors reported that PPAR-α expression was higher in rats treated with VSL#3 (a preparation composed of multi strain probiotics) compared with rats fed a HFD.

Carnitine palmitoyl transferase enzyme 1 is one of the activators of PPAR-α expressed in muscle and liver. CPT-1 promotes the uptake of fat and mitochondrial fatty acid oxidation (Kersten et al., 1999). This PPAR-α agonist increases oxidation and the amount of fatty acids available for triglyceride synthesis (Bocher et al., 2002). However, our data did not show differences in the expression of CPT-1. This finding suggests that ß-oxidation may be modulated in a PPAR-α–independent manner (Louet et al., 2001).

SREBP is involved in hepatic cholesterol homeostasis. In the liver, three isoforms (SREBP-1a, SREBP-1c, and SREBP-2) regulate lipoproteins and bile synthesis. They are also involved in the expression of over 30 genes that regulate the synthesis and use of cholesterol, fatty acids, triglycerides, and phospholipids (Kojima and Degawa, 2006; Reddy and Rao, 2006; Ahmed and Byrne, 2007).SREBP-1c is directly associated with lipid accumulation in NAFLD (Ahmed and Byrne, 2007; Duvnjak et al., 2007) and stimulates the translation of lipogenic genes such as those coding for FAS, ME, and glucose 6-phosphate dehydrogenase (G6PDH). Increased SREBP-1c expression increases triglyceride concentrations in the liver, leading to the development of NAFLD (Horton et al., 1998).

In our study, SREBP-1c expression was higher in the PC group compared to the NC group. This finding was expected, as PC animals received higher amounts of fat (cholesterol) in the diet. The data showed lower protein expression in the prebiotic supplementation group compared with the PC and probiotic groups. This finding indicates that supplementation with a prebiotic can modulate and reduce SREBP-1c expression in lipogenesis. The synbiotic group that showed lower gene expression than the probiotic group, indicating a potential limiting effect on the accumulation of lipid.

SREBP-1c and PPAR-α play important roles in the pathogenesis of NAFLD and the modulation of lipogenic and β-oxidation processes, respectively. Both compounds may contribute to the nutritional management of NAFLD. Studies have shown that PPAR-α activation can suppress the activation of SREBP-1c (Cherkaoui-Malki et al., 2001; Reddy, 2001). Those data support the results presented here: higher PPAR-α expression and lower SREBP-1c gene expression in SYN rats.

Few experiments using the NAFLD model have evaluated gene expression in liver tissue after prebiotic, probiotic, and synbiotic supplementation. This fact makes it difficult to compare our results with previously published studies on the effects of prebiotic and probiotic strains. NAFLD is a complex disease related to nutritional factors that alter the gut microbiota and intestinal barrier. Interactions among dietetic factors could modulate NAFLD pathogenesis and steatosis.

## Conclusion

Prebiotic and synbiotic supplementation improve hepatic alterations related to hypercholesterolemia through transcriptional changes that affect β-oxidation and lipogenesis.

## Author Contributions

CA contributed to the conception and design of the research, developed all gene analyses, to data interpretation and wrote the manuscript. PS developed RNA extraction, contributed to the conception and design of the research, to data interpretation and wrote the manuscript. DW contributed to the conception and design of the research, to data interpretation and wrote the manuscript. LdA, LdS, MR, and CG contributed to the development of experimental analyses such as animal care, nutritional supplementation and cholesterol analysis. DR is the expert in pathology who is responsible for the histological analyses procedures. OJ equally contributed to the conception, design of the research and to data interpretation. All authors critically revised the manuscript, gave their final approval, and agree to be accountable for all aspects of the described study, ensuring its integrity and accuracy.

## Conflict of Interest Statement

The authors declare that the research was conducted in the absence of any commercial or financial relationships that could be construed as a potential conflict of interest.

## References

[B1] Ahmed M. H. Byrne C. D. (2007). Modulation of sterol regulatory element binding proteins (SREBPs) as potential treatments for non-alcoholic fatty liver disease (NAFLD). *Drug Discov. Today.* 12 740–747. 10.1016/j.drudis.2007.07.009 17826687

[B2] Arjmandi B. H. Ahn J. Nathani S. Reeves R. D. (1992). Dietary soluble fiber and cholesterol affect serum cholesterol concentration, hepatic portal venous short-chain fatty acid concentrations and fecal sterol excretion in rats. *J Nutr.* 122 246–253. 1310108 10.1093/jn/122.2.246

[B3] Bäckhed F. Manchester J. K. Semenkovich C. F. Gordon J. I. (2007). Mechanisms underlying the resistance to diet-induced obesity in germ-free mice. *Proc. Natl. Acad. Sci. U.S.A.* 104 979–984. 10.1073/pnas.0605374104 17210919 PMC1764762

[B4] Bocher V. Pineda-Torra I. Fruchart J. C. Staels B. (2002). PPARs: transcription factors controlling lipid and lipoprotein metabolism. *Ann. N. Y. Acad. Sci.* 967 7–18. 10.1111/j.1749-6632.2002.tb04258.x 12079830

[B5] Buzzetti E. Pinzani M. Tsochatzis E. A. (2016). The Multiple-Hit Pathogenesis of Non-Alcoholic Fatty Liver Disease (NAFLD). *Metabolism* 65 1038–1048. 10.1016/j.metabol.2015.12.012 26823198

[B6] Cani P. D. Amar J. Iglesias M. A. Poggi M. Knauf C. Bastelica D. (2007). Metabolic endotoxemia initiates obesity and insulin resistance. *Diabetes* 56 1761–1772. 10.2337/db06-1491 17456850

[B7] Cherkaoui-Malki M. Meyer K. Cao W.-Q. Latruffe N. Yeldandi A. V. Rao M. S. (2001). Identification of Novel Peroxisome Proliferator-Activated Receptor α (PPARα) target genes in mouse liver using cDNA microarray analysis. *Gene Expr.* 9 291–304. 10.3727/000000001783992533 11764000 PMC5964950

[B8] de Wit N. J. Afman L. A. Mensink M. Müller M. (2012). Phenotyping the effect of diet on non-alcoholic fatty liver disease. *J. Hepatol.* 57 1370–1373. 10.1016/j.jhep.2012.07.003 22796155

[B9] Donnelly K. L. Smith C. I. Schwarzenberg S. J. Jessurun J. Boldt M. D. Parks E. J. (2005). Sources of fatty acids stored in liver and secreted via lipoproteins in patients with nonalcoholic fatty liver disease. *J. Clin. Investig.* 115 1343–1351. 10.1172/JCI23621 15864352 PMC1087172

[B10] Duvnjak M. Lerotić I. Barsić N. Tomasić V. Jukić L. V. Velagić V. (2007). Pathogenesis and management issues for non-alcoholic fatty liver disease. *World J. Gastroenterol.* 13 4539–4550. 10.3748/wjg.v13.i34.4539 17729403 PMC4611824

[B11] Eslamparast T. Poustchi H. Zamani F. Sharafkhah M. Malekzadeh R. Hekmatdoost A. (2014). Synbiotic supplementation in nonalcoholic fatty liver disease: a randomized, double-blind, placebo-controlled pilot study. *Am. J. Clin. Nutr.* 99 535–542. 10.3945/ajcn.113.068890 24401715

[B12] Esposito E. Iacono A. Bianco G. Autore G. Cuzzocrea S. Vajro P. (2009). Probiotics reduce the inflammatory response induced by a high-fat diet in the liver of young rats. *J. Nutr.* 139 905–911. 10.3945/jn.108.101808 19321579

[B13] FAO WHO (2017). *Joint FAO/WHO Working Group Report on Drafting Guidelines for the Evaluation of Probiotics in Food, London, Ontario, Canada.* Available at: http://www.who.int/foodsafety/fs_management/en/probiotic_guidelines.pdf [accessed April 6].

[B14] Fukui H. (2015). Gut-liver axis in liver cirrhosis: how to manage leaky gut and endotoxemia. *World J. Hepatol.* 7 425–442. 10.4254/wjh.v7.i3.425 25848468 PMC4381167

[B15] Guarner F. Khan A. G. Garisch J. Eliakim R. Gangl A. Thomson A. (2012). World gastroenterology organisation global guidelines. *J. Clin. Gastroenterol.* 46 468–481. 10.1097/MCG.0b013e3182549092 22688142

[B16] Guerra R. L. F. Prado W. L. Cheik N. C. Viana F. P. Botero J. P. Vendramini R. C. (2007). Effects of 2 or 5 consecutive exercise days on adipocyte area and lipid parameters in wistar rats. *Lipids Health Dis.* 6:16. 10.1186/1476-511X-6-16 17605802 PMC1933532

[B17] Horton J. D. Bashmakov Y. Shimomura I. Shimano H. (1998). Regulation of sterol regulatory element binding proteins in livers of fasted and refed mice. *Proc. Natl. Acad. Sci. U.S.A.* 95 5987–5992. 10.1073/pnas.95.11.5987 9600904 PMC27572

[B18] Kersten S. Seydoux J. Peters J. M. Gonzalez F. J. Desvergne B. Wahli W. (1999). Peroxisome proliferator-activated receptor Alpha mediates the adaptive response to fasting. *J. Clin. Investig.* 103 1489–1498. 10.1172/JCI6223 10359558 PMC408372

[B19] Kirpich I. A. Marsano L. S. McClain C. J. (2015). Gut-liver axis, nutrition, and non-alcoholic fatty liver disease. *Clin. Biochem.* 48 923–930. 10.1016/j.clinbiochem.2015.06.023 26151226 PMC4558208

[B20] Kojima M. Degawa M. (2006). Gender-related difference in altered gene expression of a sterol regulatory element binding protein, SREBP-2 by lead nitrate in rats: correlation with development of hypercholesterolemia. *J. Appl. Toxicol.* 26 381–384. 10.1002/jat.1138 16705668

[B21] Kok N. N. Taper H. S. Delzenne N. M. (2017). Oligofructose modulates lipid metabolism alterations induced by a fat-rich diet in rats. *J. Appl. Toxicol.* 18 47–53. 10.1002/(SICI)1099-1263(199801/02)18:1<47::AID-JAT474>3.0.CO;2-S 9526834

[B22] Leung C. Rivera L. Furness J. B. Angus P. W. (2016). The role of the gut microbiota in NAFLD. *Nat. Rev. Gastroenterol. Hepatol.* 13 412–425. 10.1038/nrgastro.2016.85 27273168

[B23] Lieber C. S. Leo M. A. Mak K. M. Xu Y. Cao Q. Ren C. (2004). Model of nonalcoholic steatohepatitis. *Am. J. Clin. Nutr.* 79 502–509. 14985228 10.1093/ajcn/79.3.502

[B24] Lin D. S. Connor W. E. (1980). The long term effects of dietary cholesterol upon the plasma lipids, lipoproteins, cholesterol absorption, and the sterol balance in man: the demonstration of feedback inhibition of cholesterol biosynthesis and increased bile acid excretion. *J. Lipid Res.* 21 1042–1052. 7462800

[B25] Lirussi F. Azzalini L. Orando S. Orlando R. Angelico F. (2007). “Antioxidant supplements for non-alcoholic fatty liver disease and/or steatohepatitis,” in *Cochrane Database of Systematic Reviews* ed. Lirussi F. (Chichester: John Wiley & Sons, Ltd) 10.1002/14651858.CD004996.pub3 PMC6513238 17253535

[B26] Lonardo A. Bellentani S. Argo C. K. Ballestri S. Byrne C. D. Caldwell S. H. (2015). Epidemiological modifiers of non-alcoholic fatty liver disease: focus on high-risk groups. *Dig. Liver Dis.* 47 997–1006. 10.1016/j.dld.2015.08.004 26454786

[B27] Louet J. F. Chatelain F. Decaux J. F. Park E. A. Kohl C. Pineau T. (2001). Long-chain fatty acids regulate liver carnitine palmitoyltransferase I Gene (L-CPT I) expression through a Peroxisome-Proliferator-Activated Receptor Alpha (PPARalpha)-independent pathway. *Biochem. J.* 354 189–197. 10.1042/bj3540189 11171094 PMC1221643

[B28] Mach T. (2006). Clinical usefulness of probiotics in inflammatory bowel diseases. *J. Physiol. Pharmacol.* 57(Suppl. 9) 23–33. 17242485

[B29] Manzoni M. S. Rossi E. A. Carlos I. Z. Vendramini R. C. Duarte A. C. Dâmaso A. R. (2005). Fermented soy product supplemented with isoflavones affected fat depots in juvenile rats. *Nutrition* 21 1018–1024. 10.1016/j.nut.2005.02.007 16157239

[B30] Minemura M. Shimizu Y. (2015). Gut microbiota and liver diseases. *World J. Gastroenterol.* 21 1691–1702. 10.3748/wjg.v21.i6.1691 25684933 PMC4323444

[B31] Moschen A. Susanne Kaser R. Tilg H. (2013). Non-alcoholic steatohepatitis: a microbiota-driven disease. *Trends Endocrinol. Metab.* 24 537–545. 10.1016/j.tem.2013.05.009 23827477

[B32] Nanji A. A. (2004). Animal models of nonalcoholic fatty liver disease and steatohepatitis. *Clin. Liver Dis.* 8 559–574. 10.1016/j.cld.2004.04.002 15331064

[B33] O’Hara A. M. Shanahan F. (2007). Mechanisms of action of probiotics in intestinal diseases. *ScientificWorldJournal* 7 31–46. 10.1100/tsw.2007.26 17221140 PMC5901104

[B34] Pfaffl M. W. (2001). A new mathematical model for relative quantification in real-time RT-PCR. *Nucleic Acids Res.* 29:e45 10.1093/nar/29.9.e45 PMC55695 11328886

[B35] Polyzos S. Kountouras J. Zavos C. (2009). Nonalcoholic fatty liver disease: the pathogenetic roles of insulin resistance and adipocytokines. *Curr. Mol. Med.* 9 299–314. 10.2174/156652409787847191 19355912

[B36] Polyzos S. A. Kountouras J. Mantzoros C. S. (2017). Adipose tissue, obesity and non-alcoholic fatty liver disease. *Minerva Endocrinol.* 42 92–108. 10.23736/S0391-1977.16.02563-3 27711029

[B37] Rabot S. Membrez M. Bruneau A. Gerard P. Harach T. Moser M. (2010). Germ-free C57BL/6J mice are resistant to high-fat-diet-induced insulin resistance and have altered cholesterol metabolism. *FASEB J.* 24 4948–4959. 10.1096/fj.10-164921 20724524

[B38] Reddy J. K. (2001). III. peroxisomal β-oxidation, PPARα, and steatohepatitis. *Am. J. Physiol. Gastrointest. Liver Physiol.* 281 G1333–G1339. 11705737 10.1152/ajpgi.2001.281.6.G1333

[B39] Reddy J. K. Hashimoto T. (2001). Peroxisomal β-oxidation and peroxisome proliferator –activated receptor α: an adaptive metabolic system. *Annu. Rev. Nutr.* 21 193–230. 10.1146/annurev.nutr.21.1.193 11375435

[B40] Reddy J. K. Rao M. S. (2006). Lipid metabolism and liver inflammation. II. fatty liver disease and fatty acid oxidation. *Am. J. Physiol. Gastrointest. Liver Physiol.* 290 G852–G858. 10.1152/ajpgi.00521.2005 16603729

[B41] Rossi E. A. Vendramini R. C. Carlos I. Z. Ueiji I. S. Squinzari M. M. Silva Júnior S. I. (2000). Effects of a novel fermented soy product on the serum lipids of Hypercholesterolemic rabbits. *Arq. Bras. Cardiol.* 74 209–216. 10.1590/S0066-782X2000000300003 10951824

[B42] Seki E. Schwabe R. F. (2017). Hepatic inflammation and fibrosis: functional links and key pathways. *Hepatology* 61 1066–1079. 10.1002/hep.27332 25066777 PMC4306641

[B43] Shibolet O. Karmeli F. Eliakim R. Swennen E. Brigidi P. Gionchetti P. (2002). Variable response to probiotics in two models of experimental colitis in rats. *Inflamm. Bowel Dis.* 8 399–406. 10.1097/00054725-200211000-00004 12454615

[B44] Stienstra R. Duval C. Müller M. Kersten S. (2007). PPARs, obesity, and inflammation. *PPAR Res.* 2007:95974. 10.1155/2007/95974 17389767 PMC1783744

[B45] Tipoe G. L. Ho C. T. Liong E. C. Leung T. M. Lau T. Y. H. Fung M. L. (2009). Voluntary oral feeding of rats not requiring a very high fat diet is a clinically relevant animal model of Non-Alcoholic Fatty Liver Disease (NAFLD). *Histol. Histopathol.* 24 1161–1169. 10.14670/HH-24.1161 19609863

[B46] Tremaroli V. Bäckhed F. (2012). Functional interactions between the gut microbiota and host metabolism. *Nature* 489 242–249. 10.1038/nature11552 22972297

[B47] Ulisse S. Gionchetti P. D’Alo S. Russo F. P. Pesce I. Ricci G. (2001). Expression of cytokines, inducible nitric oxide synthase, and matrix metalloproteinases in pouchitis: effects of probiotic treatment. *Am. J. Gastroenterol.* 96 2691–2699. 10.1111/j.1572-0241.2001.04139.x 11569697

[B48] Verdelho Machado M. Cortez-Pinto H. (2017). Diet, microbiota, obesity, and NAFLD: a dangerous quartet. *Int. J. Mol. Sci.* 17:481. 10.3390/ijms17040481 27043550 PMC4848937

[B49] Younossi Z. M. Koenig A. B. Abdelatif D. Fazel Y. Henry L. Wymer M. (2016). Global epidemiology of nonalcoholic fatty liver disease-meta-analytic assessment of prevalence, incidence, and outcomes. *Hepatology* 64 73–84. 10.1002/hep.28431 26707365

[B50] Zhang X.-G. Xu P. Liu Q. Yu C.-H. Zhang Y. Chen S.-H. (2006). Effect of tea polyphenol on cytokine gene expression in rats with alcoholic liver disease. *Hepatobiliary Pancreat. Dis. Int.* 5 268–272. 16698589

